# Supplementary material for: Prevalence and risk factors of geohelminthiasis among the rural village children in Kota Marudu, Sabah, Malaysia
Source: PLoS One. 2020 Sep 28;15(9):e0239680. doi: 10.1371/journal.pone.0239680 (PMC7521721; doi:10.1371/journal.pone.0239680)
Supplement: S1 Questinnaire — (PDF) [file pone.0239680.s001.pdf]

Supporting file S1: Questionnaire

Prevalensi dan Faktor Risiko Persekitaran dan Sosial Berkaitan Infeksi Cacing di kalangan Komuniti Luar Bandar di Utara Sabah

Prevalence and Associated Environmental and Social Risk Factors of Worm Infection among Rural Communities of Northern Sabah

ID Isi Rumah / *Household ID*: ______________________

Soal-Selidik / *Questionnaire*

A- Maklumat Respondent / *Particulars of Respondent*

A1. **Umur** / *Age*: __________ A2. **Jantina** / *Gender*: P / *F* ☐ L / *M* ☐

A3. **Berapa lama tinggal di kampung** / *How long residing in the kampung*: _____________

A4. **Nama Kampung** / *Name of Village*: ____

A5. **Daerah** / *District* : ☐ Kota Marudu ☐ Lain-lain/*Others*: ________________

A6. **Bangsa** / *Ethnicity*: ________________ A9. **Agama** / *Religion*: _________________

A7. **Pendidikan** / *Education*:
- ☐ Tidak pernah bersekolah / *None*
- ☐ Tadika / *Pre-school*
- ☐ Rendah / *Primary*
- ☐ Menengah / *Secondary*

A8. **Lokasi tempat sekolah anda** / *Location of your school:*
- ☐ Tidak berkenaan / *Not applicable*
- ☐ Di kawasan rumah / *in the house*
- ☐ Di kawasan atau dekat dengan kampong / *In or near the village*
- ☐ Di kawasan atau dekat dengan kampung lain dalam daerah yang sama / *In or near other village in the same district*
- ☐ Di luar daerah, dalam negeri Sabah / *Out of district, in the state of Sabah*
- ☐ Di luar Sabah / *Out of Sabah*
- ☐ Seberang laut / *Overseas*
- ☐ Lain-lain, sila nyatakan / *Others, pls specify*: _______________________________

(Nama sekolah / *Name of School*:____)

A9. **Berapa orang yang tinggal dalam rumah anda** / *How many people staying in your house?:* ____

A10. **Taraf pendidikan ibu** / *Mother's level of education*:
- ☐ Tidak pernah bersekolah / *None*
- ☐ Tadika / *Pre-school*
- ☐ Rendah / *Primary*
- ☐ Menengah / *Secondary*
- ☐ Maktab atau Universiti / *College or University*

Kemaskini pada 20150727

A11. **Pekerjaan utama ibubapa**/*Main Occupation of Parents:*

**Bapa** / *Father*	**Ibu** / *Mother*
☐ Tidak bekerja / *Not working*	☐ Tidak bekerja / *Not working*
☐ Petani / *Farmer*	☐ Petani / *Farmer*
☐ Penoreh getah / *Rubber Tapper*	☐ Penoreh getah / *Rubber Tapper*
☐ Pekerja ladang kelapa sawit / *Palm oil plantation worker*	☐ Pekerja ladang kelapa sawit / *Palm oil plantation worker*
☐ Nelayan / *Fisherman*	☐ Nelayan / *Fisherman*
☐ Suri rumah / *Housewife*	☐ Suri rumah / *Housewife*
☐ Memburu (hutan) / *hunting (forest)*	☐ Memburu (hutan) / *hunting (forest)*
☐ Kontraktor / *Construction/contractor*	☐ Kontraktor / *Construction/contractor*
☐ Pekedai / *Shopkeeper*	☐ Pekedai / *Shopkeeper*
☐ Kerajaan / *Government staff*	☐ Kerajaan / *Government staff*
☐ Swasta / *Private staff*	☐ Swasta / *Private staff*
☐ Pesara / *Retired*	☐ Pesara / *Retired*
☐ Pelajar / Student	☐ Pelajar / Student
☐ Lain-lain / *Others, pls specify* ____________________	☐ Lain-lain / *Others, pls specify* ____________________

A12. Jenis **pekerjaan sampingan lain ibubapa anda** / *Parent's other side occupation*:
Bapa/*Father*:____
Ibu/*Mother*:____

A13. **Tempat pekerjaan ibubapa anda** / *Parent's place of occupation:*

Sila tandakan (✓) pada ruang yang berkenaan	**Bapa /** *Father*	**Ibu /** *Mother*
Di kawasan rumah / *in the house*		
Di kawasan atau dekat dengan kampung / *In or near the village*		
Di kawasan atau dekat dengan kampung lain dalam daerah yang sama / *In or near other village in the same district*		
Di luar daerah, dalam negeri Sabah / *Out of district, in the state of Sabah*		
Di luar Sabah / *Out of Sabah*		
Seberang laut / *Overseas*		
Lain-lain, sila nyatakan / *Others, pls specify*:		

A14. **Jumlah pendapatan bulanan isi rumah**/*Total monthly household income*

☐ Tiada pendapatan / *No income* ☐ < RM500
☐ RM501-1000 ☐ RM1001-2000
☐ RM2001-3000 ☐ RM3001-4000
☐ RM4001-5000 ☐ > RM5001

Kemaskini pada 20150727

B- Aset / *Assets*

B1. **Tuan punya rumah** / *Household owner*
- ☐ Individu / *Individual*
- ☐ Kerajaan / *Government*
- ☐ Swasta / *Private*
- ☐ Lain-lain / *Others* (Sila nyatakan / *Pls specify*:_____________________________)

B2. **Rumah disambung dengan eletrik** / *House connected to electricity?* ☐ Ya ☐ Tidak

B3. **Jenis atap rumah anda** / *Type of roof of your house:*
- ☐ Zink /*corrugated iron*
- ☐ Simen (konkrit) / *Cement (concrete)*
- ☐ Plastik / *Plastic (PVC) sheet*
- ☐ Spandex
- ☐ Aluminium
- ☐ Kayu / *Wood*
- ☐ Rumbia / *Palm*
- ☐ Buluh / *Bamboo*
- ☐ Lain-lain / *Other* (Sila nyatakan/*pls specify*: _______________________________)

B4. **Jenis dinding rumah anda** / *Type of wall of your house:*
- ☐ Papan kayu / *Wood plank*
- ☐ Simen (konkrit) / *Cement (concrete)*
- ☐ Tanah liat / *Earth*
- ☐ Jubin / *Tiles*
- ☐ Batu-Bata / *Bricks*
- ☐ Buluh / *Bamboo*
- ☐ Lain-lain / *Other* (Sila nyatakan/*pls specify*: _______________________________)

B5. **Jenis lantai rumah anda** / *Type of floor of your house:*
- ☐ Papan kayu / *Wood plank*
- ☐ Simen (konkrit) / *Cement (concrete)*
- ☐ Tanah liat / *Earth*
- ☐ Jubin / *Tiles*
- ☐ Batu-Bata / *Bricks*
- ☐ Buluh / *Bamboo*
- ☐ Lain-lain / *Other* (Sila nyatakan/*pls specify*: _______________________________)

B6. **Jenis rumah anda** / *Type of your house:*
- ☐ Rumah satu tingkat di atas tanah / *One-storey house on the ground*
- ☐ Rumah bertiang satu tingkat / *One-storey house with stilt*
- ☐ Rumah dua tingkat / *Double-storey house*
- ☐ Lain-lain / *Other* (Sila nyatakan/*pls specify*: _______________________________)

Prevalensi dan Faktor Risiko Persekitaran dan Sosial Berkaitan Infeksi Cacing di kalangan Komuniti Luar Bandar di Utara Sabah

Prevalence and Associated Environmental and Social Risk Factors of Worm Infection among Rural Communities of Northern Sabah

C- Air minuman dan Sanitasi / Drinking water and Sanitation

C1. Apakah **punca utama bekalan air minuman** untuk ahli-ahli dalam seisi rumah anda?
What is the main source of drinking-water for members of your household?

- ☐ Air paip dalam rumah / *Piped water into dwelling*
- ☐ Air paip luar rumah / *Piped water to yard/plot*
- ☐ Paip awam / *Public tap (standpipe)*
- ☐ Perigi gali yang dilindungi */ Protected dug well*
- ☐ Perigi gali yang tidak dilindungi / *Unprotected dug well*
- ☐ Sungai / *River (stream)*
- ☐ Air Hujan / *Rainwater*
- ☐ Air botol / *Bottled water*
- ☐ Lori tangki / *Tanker-truck*
- ☐ Kolam / *Pond*
- ☐ Air Graviti / *Gravity water*
- ☐ Lain-lain / *Other* (Sila nyatakan / *pls specify*:_______________________________)

C2. Apakah **punca utama air yang digunakan untuk tujuan lain seperti memasak dan membasuh tangan**?
What is the main source of water used by your household for other purposes, such as cooking and hand washing?

- ☐ Air paip dalam rumah / *Piped water into dwelling*
- ☐ Air paip luar rumah / *Piped water to yard/plot*
- ☐ Paip awam / *Public tap (standpipe)*
- ☐ Perigi gali yang dilindungi */ Protected dug well*
- ☐ Perigi gali yang tidak dilindungi / *Unprotected dug well*
- ☐ Sungai / *River (stream)*
- ☐ Air Hujan / *Rainwater*
- ☐ Air botol / *Bottled water*
- ☐ Lori tangki / *Tanker-truck*
- ☐ Kolam / *Pond*
- ☐ Air Graviti / *Gravity water*
- ☐ Lain-lain / *Other* (Sila nyatakan / *pls specify*:_______________________________)

C3. **Apakah yang anda biasa lakukan untuk memastikan air selamat untuk diminum?**
What do you usually do to the water to make it safer to drink?

- ☐ Didih / *Boil*
- ☐ Tambah bahan peluntur (klorin) / *Add bleach (chlorine)*
- ☐ Tapis melalui kain / *Strain it through a cloth*
- ☐ Penapis air / *water filter*
- ☐ Pembasmi kuman solar / *Solar disinfection*
- ☐ Biarkan saja / *let it stand and set*
- ☐ Lain-lain / *Other* (Sila nyatakan / *pls specify*:_______________________________)

Kemaskini pada 20150727

C4. **Adakah anda biasanya membasuh tangan dahulu sebelum makan?**
Do you usually wash your hands first before eating? ☐ Ya ☐ Tidak

C4a. Adakah anda biasanya **membasuh tangan menggunakan sabun** sebelum makan?
Do you usually wash your hands using soap before eating? ☐ Ya ☐ Tidak

C5. Adakah anda selalu makan **buah mentah**?
*Do you usually eat **raw fruits**?* ☐ Ya ☐ Tidak

	Jika ya, sila jawab soalan C5a-c:<br>*If yes, please answer question C5a-c:*	Ya<br>*Yes*	Tidak<br>*No*
C5a	Adakah anda biasanya makan buah mentah yang jatuh di atas tanah?<br>*Do you usually eat raw fruits falling on the ground?*		
C5b	Adakah buah mentah dicuci terlebih dahulu sebelum dimakan?<br>*Are the raw fruits washed before consumption?*		
C5c	Boleh anda nyatakan buah mentah itu? *Can you specify the raw fruits?*		

C6. Adakah anda selalu makan **sayur/tumbuhan aquatik mentah** (termasuk ulam-ulaman) / *Do you usually eat raw vegetables/aquatic plants (including ulam)?* ☐ Ya ☐ Tidak

	Jika **ya**, sila jawab soalan C6a-f sahaja:<br>*If yes, please answer question C6a-f only:*	**Ya**<br>*Yes*	**Tidak**<br>*No*
C6a	Adakah anda biasanya makan sayur mentah yang dikutip terus dari kebun? *Do you usually eat raw vegetables harvest directly from garden?*		
C6b	Adakah anda biasanya makan sayur mentah yang jatuh di atas tanah?<br>*Do you usually eat raw vegetables falling on the ground?*		
C6c	Adakah anda makan tumbuhan akuatik mentah dari sungai/kolam?<br>Do you eat raw aquatic plants from the river/pond?		
C6d	Adakah sayur/tumbuhan akuatik mentah dicuci terlebih dahulu sebelum dimakan?<br>*Are the raw vegetable/aquatic plants washed before consumption?*		
C6e	Bagaimana cara sayur/tumbuhan aquatik mentah itu dicuci?<br>*How is the raw vegetables/aquatic plants washed?*		
C6f	Boleh anda nyatakan sayur/tumbuhan akuatik mentah itu?<br>*Can you specify the raw vegetables/raw aquatic plants?*		
	Jika **tidak**, sila jawab soalan C6g-j sahaja:<br>*If yes, please answer question C6g-j only:*		
C6g	**Jika masak, bagaimanakah sayur atau tumbuhan akuatik mentah itu dimasak?**<br>*If cook, how is the raw vegetable/aquatic plant cooked?*		
C6h	Adakah sayur/tumbuhan akuatik mentah **dicuci terlebih dahulu** sebelum dimasak? *Are the raw vegetable/aquatic plants washed before cooked?*		
C6i	Bagaimana cara sayur/tumbuhan aquatik mentah itu dicuci?<br>*How is the raw vegetables/aquatic plants washed?*		
C6j	Boleh anda nyatakan sayur atau tumbuhan akuatik itu?<br>*Can you specify the vegetables or aquatic plants?*		

Kemaskini pada 20150727

7. **Adakah anda selalu makan daging mentah?** ☐ Ya ☐ Tidak
Do you usually eat raw meat?

	Jika **ya**, sila jawab soalan C7a-d sahaja:<br>*If yes, please answer question C7a-d only:*	**Ya**<br>*Yes*	**Tidak**<br>*No*
C7a	Adakah daging mentah itu dicuci terlebih dahulu sebelum dimakan?<br>*Is the raw meat washed before consumption?*		
C7b	Bagaimana daging mentah itu dicuci sebelum dimakan?<br>*How is the raw meat washed before eaten?*		
C7c	Apakah jenis daging mentah yang anda makan?<br>*What type of raw meat do you eat?*		
C7d	Di manakah anda mendapatkan daging mentah itu?<br>*Where do you obtain the raw meat from?*		
	Jika **tidak**, sila jawab soalan C7e-i sahaja:<br>*If yes, please answer question C7e-i only:*		
C7e	**Jika masak, bagaimanakah daging mentah itu dimasak?**<br>*If cook, how is the raw meat cooked?*		
C7f	Adakah daging mentah itu **dicuci terlebih dahulu sebelum dimasak**?<br>*Is the raw meat washed before cooked?*		
C7g	Bagaimana daging mentah itu dicuci sebelum dimasak?<br>*How is the raw meat washed before cooked?*		
C7h	Apakah jenis daging yang anda masak dan makan?<br>*What type of meat do you cooked and eat?*		
C7i	Di manakah anda mendapatkan daging itu?<br>*Where do you obtain the meat from?*		

C8. **Bagaimana makanan anda dimasak?** / *How is your food cooked?*

____

C9a. Apakah **jenis kemudahan tandas yang ahli dalam isi rumah biasanya gunakan**?
What kind of toilet facility do members of your household usually use?

- ☐ Tandas tarik bersambungan dgn tangki septik / *Flush toilets connected to sewer or septic tank*
- ☐ Tandas tarik tanpa tangki septik / *Flush toilets without septic tank*
- ☐ Tandas curah bersambungan dengan tangki septik / *Pour flush connected to septic tank*
- ☐ Tandas curah tanpa tangki septik / *Pour flush without to septic tank*
- ☐ Tangki septik / *septic tank*
- ☐ Lubang tandas / *pit latrine*
- ☐ Lubang tandas dengan tapak / *Pit latrine with slab*
- ☐ Lubang tandas tanpa tapak (terbuka) / *Pit latrine without slab (open pit)*
- ☐ Tandas Kompos / *Composting toilet*
- ☐ Baldi / *Bucket*
- ☐ Tandas gantung / *Hanging toilet*

- ☐ Tiada tandas atau semak/padang / *No facilities or bush/field*
- ☐ Dalam sungai/kolam / *In the river/pond*
- ☐ Lain-lain / *Other* (Sila nyatakan / *pls specify*:____________________________)

C9b. Bolehkah orang awam menggunakan tandas ini / *Can any member of the public use this toilet?* ☐ Ya ☐ Tidak

C9c. Adakah anda kongsi kemudahan ini dengan isi rumah lain? *Do you share this facility with other households?* ☐ Ya ☐ Tidak

C10. **Adakah anda membasuh tangan setiap kali selepas buang air besar**?
Do you usually wash your hands every time after defecation? ☐ Ya ☐ Tidak

C10a. Adakah anda biasanya **membasuh tangan menggunakan sabun** selepas membuang air besar? *Do you usually wash your hands using soap every time after defecation?* ☐ Ya ☐ Tidak

C11. **Dimanakah anda biasanya membuang sampah dari rumah?**
Where do you usually dispose household waste?

- ☐ Sungai/kolam / *River/pond*
- ☐ Di sekitar luar rumah / *Around the yard outside the house*
- ☐ Tapak pelupusan di kampung / *Landfill in the village*
- ☐ Bakar / *Burn (incinerate)*
- ☐ Kitar-semula / *recycle*
- ☐ Kompos / *Compose*
- ☐ Lori sampah / *Garbage truck*
- ☐ Lubang di belakang/tepi rumah / *Hole behind/beside the house*
- ☐ Lain-lain / *Other* (Sila nyatakan / *pls specify*:____________________________)

D- Pemeliharaan haiwan, hak milik dan perhubungan

Animal rearing, ownership and contact

D1. Adakah di rumah anda mempunyai **haiwan domestik** berikut:
Does your household own any domestic animal as follows:

	Jenis haiwan / *Types of Animals*	**Ya** *Yes*	**Bilangan** *Nos*	**Tidak** *No*
D1a	Anjing / *Dogs*			
D1b	Kucing / *Cats*			
D1c	Lembu / *Cattle*			
D1d	Kerbau / *Buffalo*			
D1e	Kambing / *Goat (sheep)*			
D1e	Babi / *Pigs*			
D1f	Ayam/Itik / *Chickens/Ducks (poultry)*			
D1g	Lain-lain / *Others* (sila nyatakan/*Pls specify*) :			

Kemaskini pada 20150727

D2. Adakah terdapat **haiwan dari rumah lain/jalan yang datang masuk ke rumah/perkarangan rumah anda?**
Do animals from other households/street come into your house/yard?
☐ Ya ☐ Tidak ☐ Tidak pasti

D2a. Jika ya, berapa kekerapannya / *If yes, how frequently?*
- ☐ Tiap-tiap hari / *everyday*
- ☐ Mingguan / *weekly*
- ☐ Beberapa kali dalam sebulan / *few times each month*
- ☐ Beberapa kali dalam setahun / *Several times a year*

D3. **Jenis baja yang digunakan untuk berkebun/***Type of fertilizer used for gardening ?*
(Jika tiada kaitan, sila terus ke Soalan D4 / ***If not applicable, pls go to Question D4*****)**
- ☐ Tinja Manusia / *Human excreta*
- ☐ Baja haiwan / *Animal manure*
- ☐ Baja kimia / *Chemical*
- ☐ Lain-lain / *Other* (Sila nyatakan / *pls specify*:________________________)

D3a. Jika baja haiwan digunakan, nyatakan daripada jenis spesies
*Pls specify which species:*__________________________________

D3b. Berapa kekerapannya/*How frequently?*
- ☐ Tiap-tiap hari / *Everyday*
- ☐ Beberapa kali dalam seminggu / *Several times in a week*
- ☐ Beberapa kali dalam sebulan / *Several times in each month*
- ☐ Beberapa kali dalam setahun / *Several times a year*

D3c. Bagaimana anda menggunakan baja haiwan itu?
How do you treat the manure?
____

Kemaskini pada 20150727

**D4**	**Jika berkaitan, sila tandakan (✓) jika YA atau (X) jika TIDAK**<br>*Please tick* **(✓)** *if YES or (X) if NO, if applicable.*	**Anjing**	**Kucing**	**Lembu**	**Kerbau**	**Kambing**	**Babi**	**Ayam/ Itik**	**Lain-lain**
D4a	Biasanya dapat penjagaan veterinary<br>*Usually gets veterinary care*								
D4b	Dibenarkan dalam rumah<br>*Allowed inside the house*								
D4c	Dibenarkan berkeliaran di dalam kampung<br>*Allowed to roam in the village*								
D4d	Hanya disimpan di hadapan/belakang rumah sahaja<br>*Kept only in the front/backyard*								
D4e	Membasuh tangan selepas bermain /sentuh dengan haiwan<br>*Wash hands after playing/touch with animals*								

E- Amalan dan faktor risiko berkaitan

Habits/Practices and associated risk factors

E1. **Jenis kasut yang biasanya anda pakai di luar rumah?**
Type of footwear you usually use outside the house?

- ☐ Selipar / *slipper*
- ☐ Kasut / *shoes*
- ☐ But / *boot*
- ☐ Capal / *sandal*
- ☐ Lain-lain / *others* (Sila nyatakan / *pls specify*:________________________)

E2. **Adakah anda biasanya berkaki ayam di luar rumah ?**
Do you always walk barefooted outside the house?

☐ Ya ☐ Tidak ☐ Tidak pasti

E2a. Jika ya, berapa kekerapannya / *If yes, how frequently?*

- ☐ Tiap-tiap hari / *Everyday*
- ☐ Beberapa kali dalam seminggu / *Several times in a week*
- ☐ Beberapa kali dalam sebulan / *Several times in each month*
- ☐ Beberapa kali dalam setahun / *Several times a year*

E3. **Adakah anda membasuh kaki terlebih dahulu sebelum masuk ke dalam rumah?**
Do you wash your feet first before entering the house?

☐ Ya ☐ Tidak ☐ Tidak pasti

E3a. Jika ya, berapa kekerapannya / *If yes, how frequently?*

- ☐ Tiap-tiap hari / *Everyday*
- ☐ Beberapa kali dalam seminggu / *Several times in a week*
- ☐ Beberapa kali dalam sebulan / *Several times in each month*
- ☐ Beberapa kali dalam setahun / *Several times a year*

E4. **Adakah anda mempunyai kuku panjang?**
Do you have untrimmed, long nails?

☐ Ya ☐ Tidak ☐ Tidak pasti

E4a. **Adakah anda mempunyai kuku yang kotor?**
Do you have dirty nails?

☐ Ya ☐ Tidak ☐ Tidak pasti

E4b. **Berapa kekerapan anda memotongnya?**
How frequently do you trim your nails?

- ☐ Tiap-tiap hari / *Everyday*
- ☐ Beberapa kali dalam seminggu / *Several times in a week*
- ☐ Beberapa kali dalam sebulan / *Several times in each month*
- ☐ Beberapa kali dalam setahun / *Several times a year*

E5. **Adakah anda mempunyai amalan memakan tanah (Geophagy)?**
Do you have eating soil habit?
☐ Ya ☐ Tidak ☐ Tidak pasti

E5a. Jika ya, berapa kekerapannya / *If yes, how frequently?*
- ☐ Tiap-tiap hari / *Everyday*
- ☐ Beberapa kali dalam seminggu / *Several times in a week*
- ☐ Beberapa kali dalam sebulan / *Several times in each month*
- ☐ Beberapa kali dalam setahun / *Several times a year*

E6. **Adakah anda merokok atau pernah merokok?**
Are you a smoker?
☐ Ya ☐ Tidak ☐ Tidak pasti

E6a. Jika ya, berapa batang rokok yang anda gunakan dalam satu hari? *If yes how many cigarettes do you smoked per day?*
- ☐ 1-10
- ☐ 11 – 20
- ☐ 20 - 50
- ☐ Lebih daripada 50 / *More than 50*

E7	**Aktiviti harian yang anda lakukan.** *Daily activities*	**Tandakan/** *Tick* (✓)	**Kekerapan** *Frequency*	**Pakai kasut?** *With footwear?*	**Tidak pakai kasut?** *Not with footwear?*
a	Mandi dalam sungai atau kolam *Taking bath in the river or pond*				
b	Membasuh baju dalam sungai atau kolam *Washing clothes in the river or pond*				
c	Memburu *Hunting*				
d	Menangkap ikan *Fishing*				
e	Bercucuk tanam/bekerja di ladang *Gardening/working in the farm*				
f	Bermain bola/bersukan di padang *Playing ball/sports in the field*				
g	Berada di sekolah *In the school*				
h	Goyong-royong membersihkan ladang *Farm cleaning*				
i	Memelihara binatang ternakan *Rearing domestic animals*				
j	Menebang pokok/bertukang *Cutting down of trees/carpentry*				

Prevalensi dan Faktor Risiko Persekitaran dan Sosial Berkaitan Infeksi Cacing di kalangan Komuniti Luar Bandar di Utara Sabah

Prevalence and Associated Environmental and Social Risk Factors of Worm Infection among Rural Communities of Northern Sabah

E8. Adakah anda pernah mengambil ubat rawatan cacing?
Do you consume deworming tablet?
☐ Ya ☐ Tidak ☐ Tidak pasti

E8a. Jika ya, bilakah kali terakhir anda mengambilnya?
If yes, when did you last consume?
☐ < 6 bulan / *< 6 months*
☐ 6-12 bulan / *6-12 months*
☐ > 1 tahun / *> 1 year*
☐ Tidak pernah / *Never*

E8b. Di manakah anda mengambilnya / *Where did you consume it*?
☐ Di hospital / *In the hospital*
☐ Di klinik / *In the clinic*
☐ Di rumah / *In the house*
☐ Lain-lain / *Others*: (Nyatakan */Pls specify)* ___________________________

E8c. Adakah anda mengambil ubat atas nasihat doktor?
Did you consume the tablet based on doctor's advice?
☐ Ya ☐ Tidak ☐ Tidak pasti

Komen Tambahan / ***Additional comments***

~ TERIMA KASIH ~

Kemaskini pada 20150727

Appendix B: Information Sheet and Consent Form

Prevalensi dan Faktor Risiko Persekitaran dan Sosial Berkaitan Infeksi Cacing di kalangan Komuniti Luar Bandar di Utara Sabah

Prevalence and Associated Environmental and Social Risk Factors of Worm Infection among Rural Communities of Northern Sabah

Fakulti Perubatan dan Sains Kesihatan, Universiti Malaysia Sabah (UMS),
Jalan UMS, 88400 Kota Kinabalu, Sabah, Malaysia

RISALAH MAKLUMAT PESERTA DAN
BORANG PERSETUJUAN atau KEIZINAN PESERTA
Versi Tarikh: 15 Julai 2015

Risalah ini adalah untuk memberikan maklumat dan bertanyakan samada anda bersetuju untuk menyertai kajian mengenai **Prevalensi dan Faktor Risiko Persekitaran dan Sosial berkaitan Infeksi Cacing di kalangan Komuniti Luar Bandar di Utara Sabah**.

Terdapat beberapa cacing parasit menjangkiti manusia yang telah direkodkan di seluruh dunia, termasuk Malaysia di mana Sabah dan Sarawak adalah tidak dikecualikan. Fokus kajian ini adalah pada cacing yang ditularkan melalui tanah atau S*oil-Transmitted Helminth (*STH) yang terdiri daripada *Ascaris lumbricoides,* cacing kerawit dan *Trichuris trichuria* serta *Fasciolopsis buski* dan *Taenia solium.* Semua cacing ini adalah berhubung erat di kawasan yang tiada kemudahan asas dan infrastruktur, kekurangan sanitasi dan kebersihan persekitaran di mana tanah, air dan vegetasi telah dicemari.

Maklumat dan data mengenai prevalensi dan faktor risiko cacing tersebut di atas adalah terhad dan mungkin tidak terdapat di Sabah. Oleh yang demikian, adalah penting agar prevalensi semasa infeksi cacing diakses dan mengenalpasti faktor risiko persekitaran dan sosial antara masyarakat luar bandar Sabah. Dengan mengenalpasti faktor risiko persekitaran dan sosial terhadap pendedahan dan jangkitan, kami berharap agar kesan kedua-dua faktor ini dapat ditentukan dan fahami. Justeru itu, kajian mengenai prevalensi dan faktor risiko berkaitan jangkitan cacing terbabit akan dilaksanakan di daerah Kota Marudu, Sabah, Malaysia. Kajian ini akan berlanjutan selama 2 tahun.

Anda dipilih untuk menjadi sebahagian daripada aktiviti ini. Penglibatan anda akan menyediakan maklumat yang boleh membantu mengenalpasi strategi kawalan, pencegahan dan rawatan yang diterima masyarakat.

Jika anda bersetuju untuk menyertai kajian ini, anda akan diberi satu bekas tinja plastik yang bersih, kering dan kalis bocor. Anda akan disuruh untuk membawa sampel tinja yang segar pada keesokkan harinya. Anda akan diberi arahan ringkas mengenai cara mengumpul sampel tinja dan mengelak kontaminasi. Anda juga akan disuruh untuk menyertai satu temubual menggunakan soal-selidik yang standard. Kajian soal selidik ini akan mengambil masa 15-20 minit untuk dilengkapkan dan akan dikendalikan dengan serta-merta selepas kebenaran anda. Walaupun anda bersetuju untuk menyertai sesi soal selidik ini, anda berhak untuk menarik diri pada bila-bila masa semasa sesi dijalankan sekiranya anda inginkan.

FN: ALL02_InfoSheet&ConsentForm_BM2015_Version2 Muka Surat 1

Tiada faedah individu yang mengambil bahagian dalam kajian ini tetapi maklumat yang kami kumpul akan membantu dalam meningkatkan pemahaman kita tentang faktor-faktor risiko jangkitan cacing pada manusia. Yakinilah bahawa kami tidak akan berkongsi maklumat dengan sesiapa sahaja selain daripada beberapa orang yang berkait-rapat dengan penyelidikan ini. Kami akan memastikan penyertaan anda adalah kekal sulit dan yakinilah bahawa jawapan soal-selidik akan berada dalam simpanan ketat kami. Semua dokumen akan disimpan sekurangnya-kurangnya selama lima tahun dalam kabinet berkunci dan komputer yang dilindungi kata laluan.

Anda akan menjadi salah satu daripada peserta-peserta dalam kajian ini. Penyertaan anda adalah secara sukarela dan akan berlanjutan selama enam (6) bulan. Anda adalah bebas untuk membuat keputusan samada untuk menyertai ataupun tidak. Jika anda bersetuju untuk menyertai kajian ini, sila turunkan tandatangan pada borang persetujuan dan dikembalikan kepada pihak penyelidik. Anda boleh menukar fikiran anda bila-bila masa sahaja tanpa apa-apa kesan dan semua maklumat peneyrtaan anda akan dihapuskan.

Jika anda mempunyai sebarang pertanyaan lanjut mengenai kajian ini, anda boleh menghubungi Puan Amy Lim di no telefon 013-8942788 atau email 1912alp@gmail.com.

Terima kasih kerana mempertimbangkan permintaan untuk menyertai kajian ini.

Nota:

1. Kajian ini dikendalikan oleh Puan Amy Lim, pelajar Master di bawah penyeliaan Penyelidik *Principal/Coordinating* kajian ini, Prof. Madya Dr. Chua Tock Hing di Jabatan Pathobiologi dan Diagnostik Perubatan, Fakulti Perubatan dan Sains Kesihatan, Universiti Malaysia Sabah.
2. Kajian ini telah dikaji semula dan diluluskan oleh Jawatankuasa Etika dan Penyelidikan Perubatan, Kementerian Kesihatan Malaysia yang bertanggungjawab dalam mengkaji semula dan meluluskan sebarang projek penyelidikan yang melibatkan pesakit atau orang awam.
3. Jika anda mempunyai sebarang pertanyaan berkaitan dengan hak-hak anda sebagai peserta dalam penyelidikan ini, sila hubungi: Setiausaha, Jawatankuasa Etika & Penyelidikan Perubatan, Kementerian Kesihatan Malaysia, melalui talian telefon 03-2287 4032.

Prevalensi dan Faktor Risiko Persekitaran dan Sosial Berkaitan Infeksi Cacing di kalangan Komuniti Luar Bandar di Utara Sabah

BORANG PERSETUJUAN atau KEIZINAN PESERTA

Versi Tarikh: 15 Julai 2015

Saya telah dijemput untuk menyertai kajian ini. Saya telah membaca risalah maklumat terdahulu atau ia telah dibacakan kepada saya. Saya telah menerima penerangan mengenai sifat, tujuan dan tempoh jangka masa kajian ini. Saya mempunyai peluang untuk bertanyakan soalan dan soalan yang saya tanyakan telah dijawab dengan memuaskan.

Bahawasanya, saya, ____
(nama peserta) bersetuju untuk menyertai kajian **"Prevalensi dan Faktor Risiko Persekitaran dan Sosial Berkaitan Infestasi Cacing di kalangan Komuniti Luar Bandar di Utara Sabah".**

Saya memahami bahawa:

a. penyertaan saya adalah secara sukarela. Saya mempunyai hak untuk menarik diri daripada aktiviti kajian ini pada bila-bila masa tanpa sebarang akibat dan saya bebas untuk tidak menjawab apa-apa soalan tertentu.
b. Sekiranya maklumat yang diperolehi dalam kajian ini mungkin digunakan di presentasi persidangan dan diterbitkan dalam artikel jurnal, saya tidak akan dikenalpasti. Maklumat yang berkaitan dengan saya akan kekal sulit. Jawapan saya untuk soalan kajian tidak akan dikaitkan dengan saya.
c. Saya mungkin tidak mendapat manfaat langsung daripada mengambil bahagian dalam kajian ini.
d. Nama orang yang boleh saya hubungi telah diberikan kepada saya, sekiranya saya mempunyai sebarang pertanyaan dan/atau penjelasan lanjut mengenai kajian ini.

____________________	____________________	____________________
Nama bercetak peserta	Tandatangan atau cap ibu jari peserta	Tarikh tandatangan/Cap ibu jari

Untuk saksi – peserta adalah buta huruf/kanak-kanak:
Saya telah menyaksikan pembacaan tepat borang persetujuan kepada peserta berpotensi dan individu tersebut telah diberi peluang untuk bertanyakan soalan. Saya mengesahkan bahawa individu tersebut telah diberi persetujuan secara bebas.

____________________	____________________	____________________
Nama bercetak saksi	Tandatangan saksi	Tarikh tandatangan

Persetujuan bertulis diperolehi oleh:

____________________	____________________	____________________
Nama bercetak	Tandatangan	Tarikh Tandatangan
