# Supplementary material for: Prevalence and risk factors of geohelminthiasis among the rural village children in Kota Marudu, Sabah, Malaysia
Source: PLoS One. 2020 Sep 28;15(9):e0239680. doi: 10.1371/journal.pone.0239680 (PMC7521721; doi:10.1371/journal.pone.0239680)
Supplement: S1 Table — (PDF) [file pone.0239680.s002.pdf]

Supporting file S2. Table . Distribution of geohelminth-infected children in the villages among the households with or without basic sanitation facilities.

Village	total sampled	Sanitation facility	Sample size	*Ascaris*	Hookworm	*Trichuris*	Total worm
Kg. Bintasan Darat	46	yes	34	4	1	0	5
		no	12	0	2	1	3
Kg. Bintasan Tengah	30	yes	29	0	0	0	0
		no	1	0	0	0	0
Kg. Minansad	13	yes	13	0	0	0	0
Kg. Boluot	17	yes	16	0	3	0	3
		no	1	0	0	0	0
Kg. Mangin 1	35	yes	35	1	3	0	3*
Kg. Mangin 2	23	yes	23	0	0	0	0
Kg. Korongkom	25	yes	25	2	0	1	3
Kg. Liabas	35	yes	35	0	0	2	2
Kg. Bombong 1	11	yes	11	0	0	0	0
Kg. Gana	71	yes	71	11	1	7	18*
Kg. Kinangkaban	63	yes	38	6	1	0	7
		no	25	15	0	1	15*
Kg. Patiu	23	yes	23	0	0	0	0
Kg. Sorinsim	15	yes	15	0	0	0	0

* one of the children with double geohelminth infection

r toilet facility.
