# Supplementary figures and images for: Prevalence and risk factors of geohelminthiasis among the rural village children in Kota Marudu, Sabah, Malaysia
Source: PLoS One. 2020 Sep 28;15(9):e0239680. doi: 10.1371/journal.pone.0239680 (PMC7521721; doi:10.1371/journal.pone.0239680)

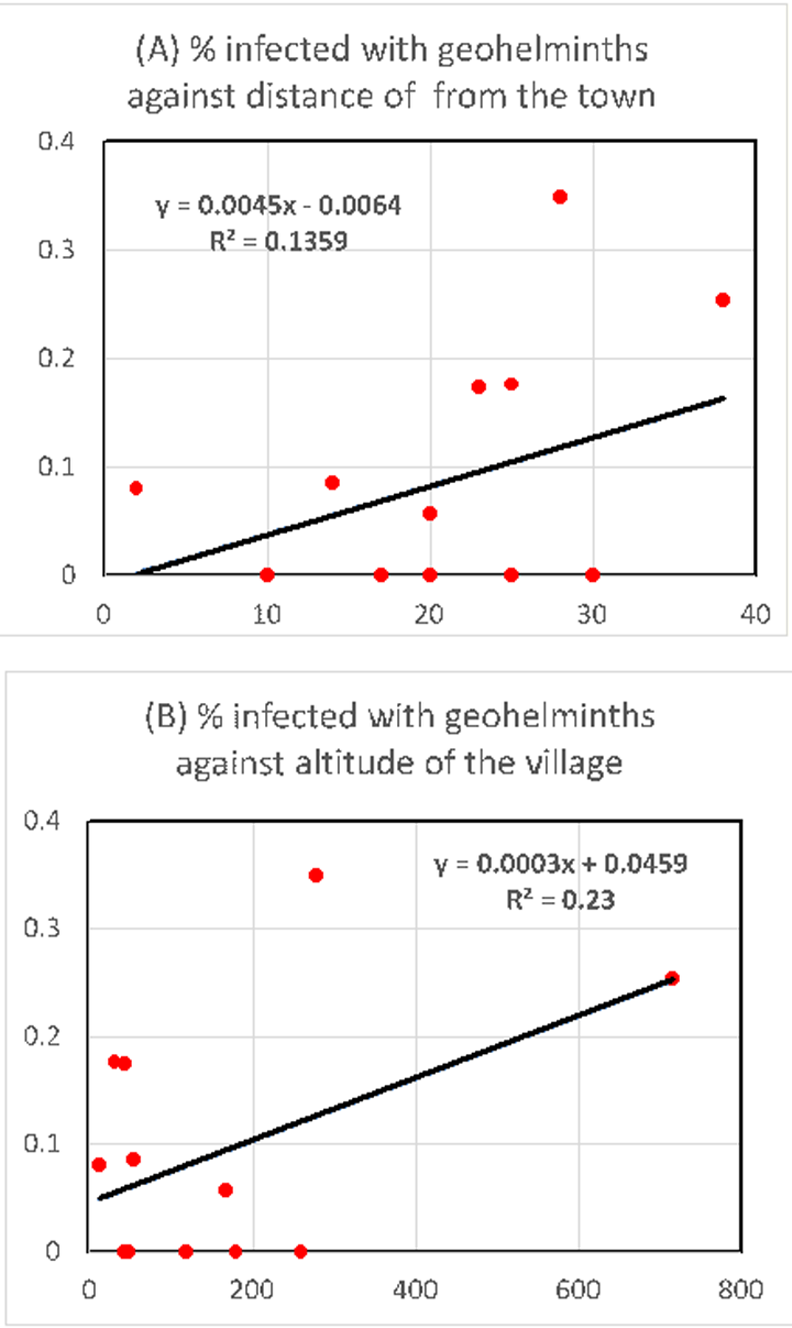

Supplement: S1 Fig — (TIF) [file pone.0239680.s003.tif]
