# Supplementary material for: Prevalence and risk factors of geohelminthiasis among the rural village children in Kota Marudu, Sabah, Malaysia
Source: PLoS One. 2020 Sep 28;15(9):e0239680. doi: 10.1371/journal.pone.0239680 (PMC7521721; doi:10.1371/journal.pone.0239680)
Supplement: Attachment — Submitted filename: Response to reviewer 8sept20.docx [file pone.0239680.s005.docx]

Response to reviewer (8 sept 2020)

Reviewer #2: My concerns raised in the earlier review have been addressed in the revised manuscript . However, a minor revision is still required. In Discussion' section- the last two paragraphs are actually 'conclusion' and/ or recommendations meant for policy makers and social/administrative bodies. These paras may be reworked so as to avoid generalized statements and give crisp/ abridged recommendations in a couple of sentences.

The 2 paragraphs have now to been reduced to one shown below:

In conclusion, this study clearly shows that geohelminthiasis still occurs with an overall prevalence of 14.3% among children of rural communities of Kota Marudu in Sabah. Infection by *Ascaris* had the highest rate at 67% compared to hookworm or *T. trichiura*. The most practical means of controlling geohelminthiasis in these communities would be expanding mass deworming programmes for school-age children, improving sanitation and water supplies, upgrading the socio-economic status and continually conducting health education programmes. The data on the prevalence of geohelminthiasis in this study would contribute to better public health monitoring and operation to reduce the infection in rural areas.
